# Mediating Effect of Communication Competence in the Relationship between Compassion and Patient-Centered Care in Clinical Nurses in South Korea

**DOI:** 10.3390/healthcare10102069

**Published:** 2022-10-18

**Authors:** Miri Jeong, Kawoun Seo

**Affiliations:** Department of Nursing, Joongbu University, Chungnam 32713, Korea

**Keywords:** communication, empathy, mediation analysis, nursing, patient-centered care

## Abstract

This study investigates the mediating effect of communication competence in the relationship between compassion and patient-centered care (PCC) in clinical nurses. We used a descriptive research approach, and our sample comprised nurses (n = 204) with more than one year of experience in patient nursing in a general hospital in South Korea. The data were collected between December 2020 and June 2021 and analyzed using descriptive statistics, *t*-tests, one-way analysis of variance, Pearson’s correlation coefficient analysis, and hierarchical multiple regression using SPSS 24.0. The Sobel test and PROCESS macro in SPSS were applied to verify the mediating effect. The mean scores for communication competence, compassion, and PCC were 3.67 ± 0.42, 64.04 ± 7.71, and 3.75 ± 0.46, respectively. Communication competence was found to partially mediate the relationship between compassion and PCC (z = 6.977, *p* < 0.001), and its explanatory power was 63.9%. To improve nurses’ PCC, developing a step-by-step and differentiated PCC improvement program that includes communication competence and compassion is necessary.

## 1. Introduction

The concept of patient-centered is derived from the concept of person-centered, which considers the situation and other interactive relationships. It implies focusing first on the subject of treatment [1]. Recently, the importance of patient-centered care (PCC) has been emphasized as the value of the medical environment that prioritizes consumer-centered care is changing. PCC is a holistic approach that respects the values and autonomy of the patient and provides professional nursing practice focusing on the individual needs of the patient [2]. In other words, it helps the participation and decision-making of the patient and provides individualized care considering the patient’s individual characteristics, needs, and preferences [2,3]. Therefore, when a patient perceives that he or she receives PCC, the intention to re-use the hospital increases, which is emerging as an important concept of medical service [4]. Previous studies have already shown that nurses’ ability to understand medical information, teamwork, clinical experience, compassion, and communication skills have an effect on patient-centered nursing [5,6]. Among them, compassion is considered to be the most important key factor in patient-centered nursing [7].

For nurses, compassion is defined as a deep awareness and strong will to alleviate the suffering of others, which not only empathizes with the patient’s difficulties but also helps form therapeutic relationships and provide quality care [8,9,10]. By understanding the physical, emotional, and spiritual difficulties of patients, nurses can become aware of their needs [11]. In other words, understanding the patient from the patient’s perspective and providing necessary nursing care is the goal of patient-centered nursing. Thus, compassion is treated as an important element in patient-centered nursing. Previous studies have already found that the empathy of nurses affects clinical performance and job satisfaction and that the empathy of nurses at general hospitals caring for cancer patients affects human-centered nursing [12,13,14]. Communication competence (CC) is essential for nurses’ compassion to lead to PCC.

Nurses must establish a good relationship with patients to provide effective nursing care. Nurses’ CC is a means for establishing a therapeutic relationship between the nurse and the patient and is an essential element for successful nursing [15]. With the recent increase in the provision of patient-centered medical care, the role of the patient in clinical decision-making is changing from a passive object that accepts the medical staff’s independent judgment to a subject who seeks and derives treatment methods together with the medical staff [16,17]. Shared decision-making is presented as a clinical decision-making process model in which the patient’s role is more active—given patient participation and influence—than the one-way medical interventionist model of medical staff. This is an enhanced new patient–caregiver decision-making model [18,19]. In shared decision-making, to formulate the best decisions together with patients, medical staff support patients’ right to know and autonomy by providing them with up-to-date evidence and information and by ensuring that the information provided is understood [20,21]. Patients collaborate with medical staff in decision-making by sharing their values, preferences, and experiences with them and choosing the appropriate treatment together [21]. Nurses’ role in shared decision-making is to encourage patients to participate in the decision-making process. This provides patients with the information they need, helps share the treatment and care plans with them, and plays a role in supporting and advocating for patients and caregivers to formulate the right decisions when deciding on treatment methods [22]. In this process, patients and caregivers believe that nurses think from their perspective and support them, which increases their trust in nurses.

As such, nurses’ compassion is an important influencing factor in patient-centered nursing, and appropriate communication competency is necessary for nurses’ compassion to be translated into patient-centered nursing performance. However, most studies conducted hitherto on patient-centered nursing for nurses have only identified factors affecting patient-centered nursing. Studies examining the role of CC in the relationship between compassion and patient-centered nursing are scarce. Therefore, in this study, we aim to prepare fundamental data for developing a program to improve nurses’ patient-centered nursing by confirming the effect of compassion on nurses’ patient-centered nursing and identifying the role of CC in the relationship between compassion and patient-centered nursing.

## 2. Methods

### 2.1. Study Design and Participants

This study was designed as a cross-sectional study to determine the mediating effect of CC on the relationship between compassion competence and patient-centered nursing. We conducted this study with clinical nurses with at least 1 year of career experience. The specific inclusion criteria were (1) nurses who took care of patients within the past six months, and (2) nurses who clearly understood the purpose of the study and agreed to participate. Nurses in departments that do not face patients or directly perform nursing, such as the operating room, anesthesia and pain medicine department, and infection control department, were excluded from this study. The appropriate sample size required for data analysis was calculated as a post hoc test for multiple linear regression analysis using the G-Power 3.1.9.7 program. For effect size, data from 186 people were used based on effect size = 0.15 (median), significance level (α) = 0.05, and 10 predictors of multiple linear regression analysis. As the power was 98.1% in the analysis, the number of samples was confirmed to be suitable for the study. Participants were recruited through convenience sampling between December 2020 and June 2021. The researcher first explained the purpose and necessity of the questionnaire to the head nurse in each department and then obtained consent to proceed with the questionnaire. Afterward, the online survey URL was distributed to the nurses who agreed to participate in the study through the head nurse, and the survey was conducted. A small gift was provided to the nurses who participated in the survey. Data were collected from a total of 207 nurses; however, data from 3 nurses who were missing or not sincere in the analysis were excluded. Thus, 204 nurses were used for the analysis.

### 2.2. Ethical Consideration

This study was approved by the Institutional Review Board of C University (IRB No. 202011-SB-146-01). After providing a sufficient explanation of the background, purpose, and confidentiality of the data to the subjects, the researcher conducted a survey for data collection only if the participants agreed to the study. It explained that all the data were anonymous and would not be used for purposes other than this study. It also explained that participation could be withdrawn at any time without penalty, if desired, even after discontinuing or completing the survey while participating in the study. The participants were remunerated a small amount to complete the questionnaire. The input and management of the collected data were performed by the researcher in charge of this study.

### 2.3. Questionnaire

The questionnaire comprised 56 items: 7 questions on the participants’ general characteristics and clinical experience, and 15, 17, and 17 items for measuring CC, compassion, and PCC, respectively.

#### 2.3.1. Communication Competence 

CC was measured using the interpersonal CC scale developed by Hur [23]. This questionnaire consisted of 15 items. Each question used a 5-point Likert scale to measure responses, and the higher the total score, the higher the CC. The reliability at the time of development [23], indicated by Cronbach’s α, was 0.91, and the reliability of the instrument in this study was 0.88.

#### 2.3.2. Compassion

Compassion was measured using the Nurses’ Compassion Scale developed by Lee and Seoumn [10]. This questionnaire consisted of 17 items. Each question used a 5-point Likert scale to measure responses, and the higher the total score, the higher the compassion. Cronbach’s α at the time of development [10], as well as that in this study, was identical (0.91).

#### 2.3.3. Patient-Centered Care

PCC was measured using the PCC competence scale for hospital nurses developed by Hwang [24]. This questionnaire comprised 17 items. Each question used a 5-point Likert scale to measure responses, and the higher the total score, the higher the PCC. The reliability at the time of development, reflected in Cronbach’s α was 0.92 [24], and that of the instrument in this study was 0.91.

### 2.4. Statistical Analysis

The research data were analyzed using SPSS/WIN 24.0. The general characteristics and measurement variables of the participants were analyzed using descriptive statistics. Differences in decision-making strategy, empathy competency, and patient-centered nursing competency according to the general characteristics of subjects were confirmed by independent *t*-tests or one-way analysis of variance, and correlations between major variables were analyzed using Pearson’s correlation coefficient. To analyze the mediating effects of decision-making competence in the relationship between compassion and PCC competence, we performed hierarchical regression analyses employing the three-step procedure of Baron and Kenny [25]. A Sobel test was performed to verify the significance of mediating effect sizes. Before the regression analysis, we confirmed that the data satisfied the basic assumptions of regression: linearity, homogeneity of variation, and normality of multicollinearity. Furthermore, Model 4 (bootstrap sample size = 5000) of Hayes’s [26] PROCESS macro for SPSS (3.3 version) with nonparametric sample extraction (bootstrapping) verified the significance of the mediated effect with a 95% confidence interval (CI).

## 3. Results

### 3.1. Sociodemographic Characteristics

The mean age was 33.63( ±6.19) years and 96.6% (n = 197) of the participants were female. More than half of the patients were single (55.4%; n = 113). Moreover, 79.4% of the participants had completed university studies and 57.4% were working in the ward (medical and surgical units). The participants were mostly staff nurses (83.3%; n = 170). The average total work experience was 101.17 (±67.01) months, while 67.2% of the nurses had more than 60 months of work experience (Table 1).

### 3.2. Differences in Levels of CC, Compassion, and PCC according to Participants’ Sociodemographic Characteristics

Table 1 presents the results of the analysis of the differences in the levels of CC, compassion, and PCC according to the participants’ sociodemographic characteristics. The compassion competence of nurses working in the emergency room was lower than that of nurses working in departments other than the intensive care unit.

### 3.3. Correlation among CC, Compassion, and PCC

As illustrated in Table 2, CC was significantly positively correlated with compassion (r = 0.67, *p* < 0.001) and PCC (r = 0.74, *p* < 0.001), and compassion had a significant positive correlation with PCC (r = 0.71, *p* < 0.001).

### 3.4. Mediating Effects of CC in the Relationship between Compassion and PCC

The results of verifying the mediating effect of CC on the relationship between compassion and PCC in nurses are shown in Table 3. The multicollinearity of the independent variables to be included in the regression model to test the assumptions of regression was greater than 0.1 with a tolerance of 0.553 to 1.000, and a variance inflation factor of 1.000 to 1.807, with less than 10 indicating no multicollinearity problem. The Durbin–Watson index was 1.781–1.867, close to 2, implying it was independent, regardless of the residuals. To verify the mediating effect of CC on the relationship between compassion and PCC, Baron and Kenny’s three-step results were as follows: In step one, compassion, an independent variable, significantly affected CC, a mediator variable (β = 0.668, *p* < 0.001; Table 3), with an explanatory power of 44.4%. In step two, compassion, an independent variable, significantly affected PCC, a dependent variable (β = 0.714, *p* ≤ 0.001), with an explanatory power of 50.7%. In step three, CC mediated and significantly affected PCC. In step 3, compassion, an independent variable, had a significant effect on PCC, a dependent variable (β = 0.390, *p* ≤ 0.001), and CC, a dependent variable, had a significant impact on PCC, a dependent variable (β = 0.484, *p* ≤ 0.001); thus, the mediating effect was verified. As the non-standardization factor (B) decreased from 0.714 in step 2 to 0.390 in step 3, it was shown to have a partial mediating effect. As a result of the verification of multiple mediators in the Sobel test, CC was confirmed as a significant mediator in the relationship between compassion and PCC (Z = 6.977, *p* < 0.001). Consequent to confirming the statistical significance of the mediating effect by applying Model 4 of the PROCESS macro, the range of the upper and lower limits of the 95% CI was 0.231 to 0.417, which was statistically significant because 0 was not included. Therefore, a mediating effect of CC in the effect of compassion on PCC was confirmed (Table 3).

## 4. Discussion

This study was conducted to provide fundamental data for developing a program to improve clinical nurses’ PCC competency by confirming the mediating effect of CC on the relationship between compassion competence and PCC.

The number of female nurses participating in this study was 96.6%. This is similar to the fact that 95.7% of active nurses were women in the 2018 health and medical personnel survey [27]. The participants of this study were mostly in their twenties (38.7%) and thirties (50%). However, in the case of active nurses in Korea, the proportion of those in their 20s and 30s was about 70%, indicating that the age of the participants of this study was rather low [27]. The average CC score of the participants in this study was 3.67 points (out of 5) and did not differ according to general characteristics. In Lee and Kim’s [6] study of intensive care unit nurses, the average CC score was 3.56, which was similar. However, in Park and Kim’s [24] study of psychiatric nurses, although the CC score was similar, with an average of 3.59, it differed as the CC varied according to marital status and religion. The reason for this can be interpreted as nurses, the subjects of the study, included all ward nurses in this study, whereas the study by Park and Kim [28] targeted psychiatric nurses who needed more communication skills. A nurse’s communication skill forms a therapeutic relationship and becomes a medium for professional interactions with patients [22]. Even when patients in the intensive care unit cannot communicate well, and the nurse attempts to communicate through various methods and explains the treatment in an easy-to-understand language, the patients expressed that they felt that the nurse was providing appropriate care [22]. As nurses’ communication is not limited to language, an educational program should be developed and implemented that considers their department’s characteristics when improving their CC.

The compassion score of the participants in this study was 3.76 (out of 5) and differed depending on the work department. This result is similar to that of Cho and Seo [29], who reported that the compassion score was 3.56 points in a study of clinical nurses. In Lee and Gang’s [30] study of nurses caring for patients with dementia, the compassion score was 3.68, which was at a similar level as that in our study. Comparing departmental compassion scores in this study, emergency room nurses had the lowest compassion level. Emergency room nurses must meet various patients daily, from severe to mild emergency patients, and provide rapid treatment according to their severity [5]. This can be thought to be because emergency room nurses aim to satisfy the nursing needs of various patients by performing prompt and accurate treatment given the nature of the emergency room. However, considering the specificity of the goals of nursing in the emergency room, research on the compassion competence of emergency room nurses is limited. As the compassion competency of nurses is an essential element for PCC pursued by modern nursing and is a major factor that increases patient satisfaction with nursing, continuous research is needed to explore and improve the compassion competency of emergency room nurses.

The PCC score of the participants in this study was 3.75 and did not differ according to general characteristics. This score is similar to the score of 3.69 for PCC in Kim and Cha’s [5] study of clinical nurses. However, their study differed as the PCC varied according to age, marital status, religion, and clinical experience. Kim and Cha argued that this was because the level of understanding of patients increases as their age increases and as they experience several interpersonal relationships due to family relationships or clinical experiences. In a study by Lee and Kim [6], the only characteristic that showed a difference in PCC was the clinical experience. However, in this study, although the age and clinical level of the participants were similar to those in previous studies, the PCC characteristics did not differ. As these characteristics can vary depending on the method of the hospital’s nursing delivery system [6], a study to compare and analyze the difference in PCC according to the hospital’s nursing delivery system should be conducted in the future.

The results of this study demonstrate a positive correlation between the compassion competency of nurses and PCC, and compassion is a direct influencing factor on nurses’ PCC. This means that nurses with high compassion competence have high PCC. PCC includes not only interventions through prescription medication or nursing processes but also listening to and empathizing with the individual needs of a nurse in caring for a patient in clinical practice [31]. In a previous study that explored the experience of PCC in intensive care unit patients, empathy appeared to be the first category of PCC [22]. This can be thought of as being unable to provide PCC as nurses cannot empathize with patients if they do not understand their plight, and if they are not compassionate, they cannot know what care patients need. Therefore, to improve the PCC in nurses, improvement in compassion competence should be prioritized. In addition, the element of compassion competence should be included in the development of PCC strengthening programs.

Nurses’ CC was positively correlated with compassion and PCC. In addition, CC had a partial mediating effect on the relationship between nurses’ compassion and PCC. This means that CC is important for nurses’ compassion to lead to PCC. Nurses provide patients with the necessary knowledge, skills, emotional support, and problem-solving methods during the treatment process. Such therapeutic communication improves satisfaction with nursing services by preventing and solving various problems that occur during the treatment process [15]. In a study by Choi and Kang [15], when inpatients demand nursing, if the nurse’s CC is good, satisfaction with nursing is improved. Therefore, CC can be an important strategy for nurses’ compassion to lead to PCC. Based on this, in nursing research, it is necessary to develop a program to improve the PCC of nurses. In the development of these programs, CC should be administered as an element of the program along with the compassion of nurses. In nursing practice, it is necessary to implement a practical competency-strengthening program to improve nurses’ compassion and at the same time improve CC. Finally, in nursing education, programs to improve the compassion and CC of nursing students should be placed in order to improve nursing students’ PCC competency.

As a limitation, generalizing the results of this study is difficult because it was conducted with only a few nurses in Korea. However, exploring the relationship between nurses’ CC, compassion, and patient-centered nursing and confirming that CC has a partial mediating effect on the effect of nurses’ compassion on patient-centered nursing would be meaningful. The results of this study can be used as fundamental data for developing programs to improve PCC for nurses in the future.

## 5. Conclusions

This study confirmed the partial mediating effect of CC on the relationship between nurses’ compassion and PCC. The results showed that the CC of nurses is a parameter that positively affects the connection between nurses’ compassion and PCC and that applying a program to strengthen compassion and CC is necessary to improve nurses’ PCC. Therefore, improving nurses’ PCC necessitates checking the level of their compassion and CC. In addition, the application of a program to increase nurses’ compassion and CC will help improve their PCC.

The results of this study have the following implications. In terms of nursing research, this study was conducted with a limited number of nurses, which limits the generalization of the research results. To improve the nurses’ PCC in terms of nursing practice, we suggest the development and application of a nursing education program that can strengthen PCC competence by assessing the level of compassion and CC of nurses. In terms of nursing education, programs, and education to improve compassion and CC for nursing students are necessary.

## Figures and Tables

**Table 1:** Characteristics of the Participants (*N* = 204).

Characteristics	Categories	n(%) or M ± SD	CC		Compassion		PCC	
			M ± SD	t or F (*p*)	M ± SD	t or F (*p*)	M ± SD	t or F (*p*)
Age		33.63 ± 6.19						
	20~30	79 (38.7)	3.65 ± 0.36	0.66 (0.520)	63.78 ± 6.81	0.40 (0.673)	3.70 ± 0.44	0.82 (0.442)
	31~40	103 (50.5)	3.67 ± 0.45		63.94 ± 8.17		3.76 ± 0.48	
	≥41	22 (10.8)	3.77 ± 0.49		65.41 ± 8.69		3.84 ± 0.50	
Gender	Male	7 (3.4)	3.63 ± 0.27	0.25 (0.801)	32.71 ± 5.88	0.46 (0.645)	3.75 ± 0.46	1.12 (0.266)
	Female	197 (96.6)	3.58 ± 0.43		64.09 ± 7.77		3.55 ± 0.60	
Marital status	Married	89 (43.6)	3.74 ± 0.46	1.82 (0.070)	63.89 ± 7.29	−0.17 (0.868)	3.79 ± 0.48	1.21 (0.229)
	Single	113 (55.4)	3.63 ± 0.39		64.07 ± 8.09		3.71 ± 0.45	
	No response	2 (1.0)						
Level of education	Junior college	22 (10.8)	3.73 ± 0.27	0.61 (0.543)	62.95 ± 6.51	1.94 (0.146)	3.80 ± 0.31	2.89 (0.058)
	University	162 (79.4)	3.66 ± 0.43		63.80 ± 7.60		3.71 ± 0.46	
	≥Graduate School	20 (9.8)	3.75 ± 0.48		67.15 ± 9.26		3.97 ± 0.56	
Unit	Medical unit ^a^	65 (31.9)	3.70 ± 0.45	1.40 (0.234)	64.29 ± 7.92	4.43 (0.002) a, b, e > c	3.74 ± 0.46	1.57 (0.184)
	Surgical unit ^b^	52 (25.5)	3.69 ± 0.34		64.81 ± 5.95		3.73 ± 0.40	
	Emergency unit ^c^	11 (5.4)	3.38 ± 0.59		55.09 ± 11.81		3.44 ± 0.62	
	Intensive care unit ^d^	9 (4.4)	3.67 ± 0.32		62.44 ± 4.69		3.75 ± 0.29	
	Others ^e^	67 (32.8)	3.69 ± 0.42		64.88 ± 7.46		3.82 ± 0.50	
Position	≥Charge nurse	34 (16.7)	3.76 ± 0.43	1.32 (0.189)	65.59 ± 8.96	1.29 (0.200)	3.84 ± 0.43	1.34 (0.182)
	Staff nurse	170 (83.3)	3.66 ± 0.42		63.73 ± 7.42		3.73 ± 0.47	
Total career (month)		101.17 ± 67.01						
	≤12	4 (2.0)	3.56 ± 0.24	0.38 (0.821)	64.00 ± 8.41	0.56 (0.694)	3.65 ± 0.37	0.32 (0.861)
	13~36	26 (12.7)	3.64 ± 0.37		62.88 ± 9.19		3.68 ± 0.42	
	37~60	37 (18.1)	3.64 ± 0.52		63.24 ± 7.52		3.71 ± 0.53	
	61~120	72 (35.3)	36.67 ± 0.42		63.90 ± 6.89		3.78 ± 0.44	
	>121	65 (31.9)	3.72 ± 0.40		65.11 ± 8.10		3.77 ± 0.49	

M = Mean; SD = Standard Deviation; CC = Communication Competence; PCC = Patient-Centered Care. Superscripts a–e indicated the results of the post-hoc analysis.

**Table 2:** Correlation among shared decision-making, compassion, and patient-centered care (*N* = 204).

Variables	M ± SD	Skewness	Kurtosis	1	2
				r (*p*)	r (*p*)
1. Communication Competence	3.67 ± 0.42	−0.14	1.40	1	
2. Compassion	64.04 ± 7.71	−0.25	1.67	0.67 (<0.001)	1
3. Patient-centered nursing	3.75 ± 0.46	−0.46	0.54	0.74 (<0.001)	0.71 (<0.001)

M = Mean; SD = Standard Deviation.

**Table 3:** Mediating effects of CC on the relationship between compassion and PCC (*N* = 204).

Step	Path	B (SE) 95% CI	*β*	T (*p*)	Adj.R^2^	F (*p*)
STEP 1	Compassion → CC	0.668 (0.052) [0.565, 0.772]	0.668	12.77 (<0.001)	0.444	163.09 (<0.001)
STEP 2	Compassion → PCC	0.714 (0.049) [0.616, 0.811]	0.714	14.476 (<0.001)	0.507	209.54 (<0.001)
STEP 3	Compassion → PCC	0.390 (0.057) [0.278, 0.503]	0.390	6.847 (<0.001)	0.639	177.56 (<0.001)
	CC → PCC	0.484 (0.057) [0.371, 0.596]	0.484	8.483 (<0.001)		

Note: Sobel test: Z = 6.977, *p* < 0.001. PROCESS macro bootstrapping 95% CI [0.231, 0.417]. CC: Communication Competence; PCC: Patient-centered Care; SE = standardized error; CI = confidence interval.
